# 1-[4-(4-Fluoro­phen­yl)-6-methyl-2-sulfanyl­idene-1,2,3,4-tetra­hydro­pyrimidin-5-yl]ethanone

**DOI:** 10.1107/S1600536812033727

**Published:** 2012-08-01

**Authors:** N. Anuradha, A. Thiruvalluvar, S. Chitra, D. Devanathan, R. J. Butcher

**Affiliations:** a PG Research Department of Physics, Rajah Serfoji Government College (Autonomous), Thanjavur 613 005, Tamilnadu, India; b Department of Chemistry, K.S.R. College of Engineering, K.S.R. Kalvi Nagar, Tiruchengode 637 215, Tamilnadu, India; c Department of Chemistry, Government Arts College, C. Mutlur 608 102, Chidambaram, Tamilnadu, India; d Department of Chemistry, Howard University, 525 College Street NW, Washington, DC 20059, USA.

## Abstract

In the title mol­ecule, C_13_H_13_FN_2_OS, the heterocyclic ring adopts a slightly distorted flattened boat conformation, and the plane through the four coplanar atoms makes a dihedral angle of 87.45 (14)° with the benzene ring. The thione, acetyl and methyl groups lie on the opposite side of the heterocyclic mean plane to the fluorophenyl group, which has an axial orientation. N—H⋯O, N—H⋯S, C—H⋯F and C—H⋯O inter­molecular hydrogen bonds and a weak C—H⋯π inter­action involving the benzene ring are found in the crystal structure.

## Related literature

For chemical and biological applications of dihydro­pyrimidine derivatives and for the closely related crystal structure of the chloro derivative, see: Anuradha *et al.* (2009 ▶).
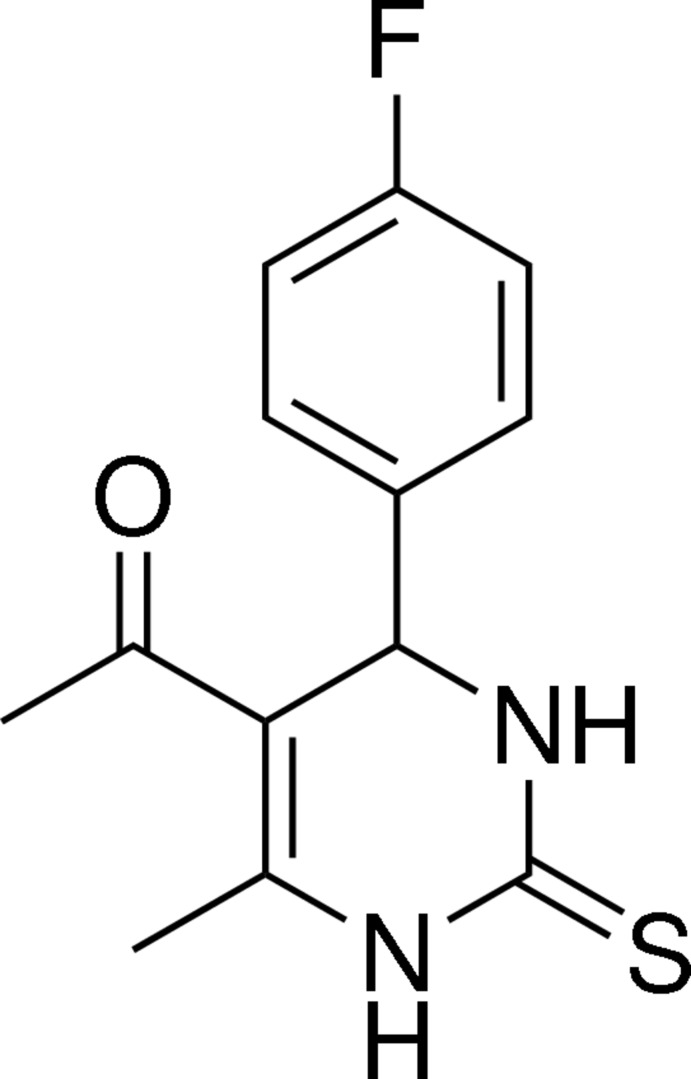


## Experimental

### 

#### Crystal data

C_13_H_13_FN_2_OS
*M* *_r_* = 264.32
Triclinic, 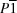

*a* = 7.1775 (11) Å
*b* = 8.1099 (13) Å
*c* = 12.490 (2) Å
α = 103.529 (15)°
β = 91.933 (14)°
γ = 106.971 (14)°
*V* = 672.0 (2) Å^3^
*Z* = 2
Cu *K*α radiation
μ = 2.17 mm^−1^
*T* = 123 K
0.59 × 0.36 × 0.06 mm

#### Data collection

Oxford Diffraction Xcalibur Ruby Gemini diffractometer
Absorption correction: analytical (*CrysAlis PRO*; Agilent, 2012 ▶) *T*_min_ = 0.515, *T*_max_ = 0.891
3949 measured reflections
2629 independent reflections
2109 reflections with *I* > 2σ(*I*)
*R*_int_ = 0.055

#### Refinement

*R*[*F* ^2^ > 2σ(*F* ^2^)] = 0.080
*wR*(*F* ^2^) = 0.233
*S* = 1.12
2629 reflections
173 parameters
H atoms treated by a mixture of independent and constrained refinement
Δρ_max_ = 0.82 e Å^−3^
Δρ_min_ = −0.37 e Å^−3^

### 

Data collection: *CrysAlis PRO* (Agilent, 2012 ▶); cell refinement: *CrysAlis PRO*; data reduction: *CrysAlis PRO*; program(s) used to solve structure: *DIRDIF2008* (Beurskens *et al.*, 2008 ▶); program(s) used to refine structure: *SHELXL97* (Sheldrick, 2008 ▶); molecular graphics: *ORTEP-3* (Farrugia, 1997 ▶) and *PLATON* (Spek, 2009 ▶); software used to prepare material for publication: *PLATON*.

## Supplementary Material

Crystal structure: contains datablock(s) global, I. DOI: 10.1107/S1600536812033727/wn2486sup1.cif

Structure factors: contains datablock(s) I. DOI: 10.1107/S1600536812033727/wn2486Isup2.hkl

Supplementary material file. DOI: 10.1107/S1600536812033727/wn2486Isup3.cdx

Supplementary material file. DOI: 10.1107/S1600536812033727/wn2486Isup4.cml

Additional supplementary materials:  crystallographic information; 3D view; checkCIF report

## Figures and Tables

**Table 1:** Hydrogen-bond geometry (Å, °) *Cg*2 is the centroid of the C41–C46 benzene ring.

*D*—H⋯*A*	*D*—H	H⋯*A*	*D*⋯*A*	*D*—H⋯*A*
N1—H1⋯O15^i^	0.82 (5)	2.10 (5)	2.867 (5)	156 (5)
N3—H3⋯S2^ii^	0.85 (6)	2.50 (6)	3.350 (4)	174 (6)
C16—H16*A*⋯F4^iii^	0.98	2.50	3.358 (6)	146
C61—H61*A*⋯F4^iii^	0.98	2.47	3.319 (6)	145
C61—H61*B*⋯O15^i^	0.98	2.54	3.383 (5)	144
C16—H16*C*⋯*Cg*2^iv^	0.98	2.92	3.633 (5)	130

Symmetry codes: (i) 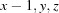; (ii) 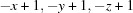; (iii) 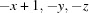; (iv) 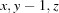.

**Fig. 1.**
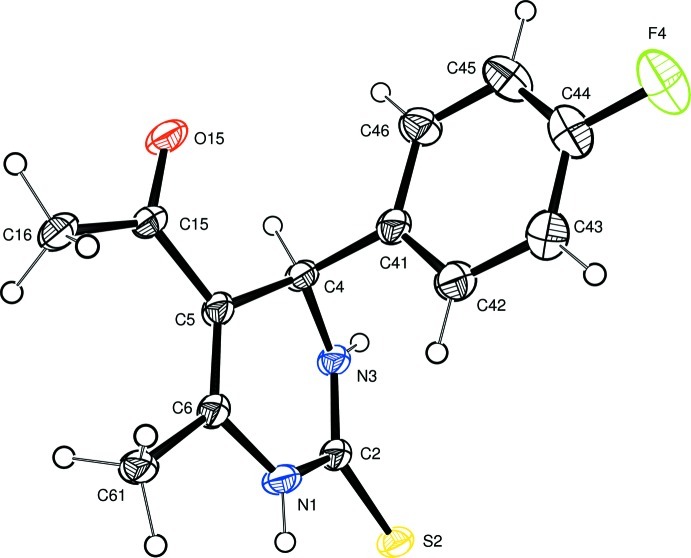
The molecular structure of the title compound, with displacement ellipsoids drawn at the 30% probability level. H atoms are shown as small spheres of arbitrary radius.

**Fig. 2.**
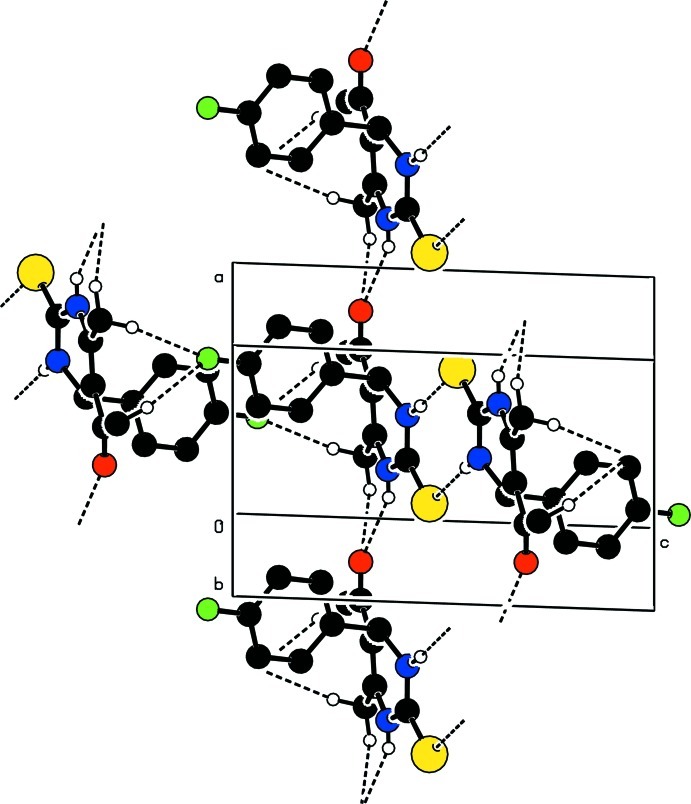
The packing of the title compound, viewed along the b axis. Dashed lines indicate hydrogen bonds. H atoms not involved in hydrogen bonding have been omitted.

## supplementary crystallographic information

### Comment

As part of our investigations of dihydropyrimidine derivatives (Anuradha *et al.*, 2009) to compare their chemical and biological activities, we have undertaken the X-ray cryatal structure analysis of the title compound.

In the title molecule, C_13_H_13_FN_2_OS (Fig.1), the heterocyclic ring adopts a slightly distorted flattened boat conformation, and the plane through the four coplanar atoms (C2,N3,C5 and C6) makes a dihedral angle of 87.45 (14)° with the benzene ring. The thione, acetyl and methyl groups have equatorial orientations and the fluorophenyl group has an axial orientation. N1—H1···O15, N3—H3···S2, C16—H16A···F4, and C61—H61B···O15 intermolecular hydrogen bonds and a weak C16—H16C···π interaction involving the benzene (C41—C46) ring are found in the crystal structure (Fig.2, Table 1).

### Experimental

A solution of acetylacetone (1.0012 g, 0.01 mol), 4-fluorobenzaldehyde (1.25 g, 0.01 mol) and thiourea (1.14 g, 0.015 mol) was heated under reflux in the presence of calcium fluoride (0.07 g, 0.001 mol) for 2 h (monitored by TLC). After completion of the reaction, the reaction mixture was cooled to room temperature and poured into crushed ice. The solid product was filtered under suction and purified by recrystallization from hot methanol to give the product in the pure form. Yield 1.92 g (96%).

### Refinement

The two N-bound H atoms were located in a difference Fourier map and refined freely; N1—H1 = 0.82 (5) Å and N3—H3 = 0.85 (6) Å. The remaining H atoms were positioned geometrically and allowed to ride on their parent atoms, with C*sp*^2^—H = 0.95, *C*(methyl)—H = 0.98, and C(methine)—H = 1.00 Å; *U*_iso_(H) = k*U*_eq_(C), where k = 1.5 for methyl and 1.2 for all other H atoms.

### Crystal data

**Table:**

C_13_H_13_FN_2_OS	*Z* = 2
*M**_r_* = 264.32	*F*(000) = 276
Triclinic, *P*1	*D*_x_ = 1.306 Mg m^−^^3^
Hall symbol: -P 1	Melting point: 509 K
*a* = 7.1775 (11) Å	Cu *K*α radiation, λ = 1.54184 Å
*b* = 8.1099 (13) Å	Cell parameters from 1186 reflections
*c* = 12.490 (2) Å	θ = 3.7–75.9°
α = 103.529 (15)°	µ = 2.17 mm^−^^1^
β = 91.933 (14)°	*T* = 123 K
γ = 106.971 (14)°	Plate, colourless
*V* = 672.0 (2) Å^3^	0.59 × 0.36 × 0.06 mm

### Data collection

**Table:**

Oxford Diffraction Xcalibur Ruby Gemini diffractometer	2629 independent reflections
Radiation source: Enhance (Cu) X-ray Source	2109 reflections with *I* > 2σ(*I*)
Graphite monochromator	*R*_int_ = 0.055
Detector resolution: 10.5081 pixels mm^-1^	θ_max_ = 76.1°, θ_min_ = 3.7°
ω scans	*h* = −7→8
Absorption correction: analytical (*CrysAlis PRO*; Agilent, 2012)	*k* = −8→10
*T*_min_ = 0.515, *T*_max_ = 0.891	*l* = −15→11
3949 measured reflections	

### Refinement

**Table:**

Refinement on *F*^2^	Primary atom site location: structure-invariant direct methods
Least-squares matrix: full	Secondary atom site location: difference Fourier map
*R*[*F*^2^ > 2σ(*F*^2^)] = 0.080	Hydrogen site location: inferred from neighbouring sites
*wR*(*F*^2^) = 0.233	H atoms treated by a mixture of independent and constrained refinement
*S* = 1.12	*w* = 1/[σ^2^(*F*_o_^2^) + (0.1003*P*)^2^ + 1.2879*P*] where *P* = (*F*_o_^2^ + 2*F*_c_^2^)/3
2629 reflections	(Δ/σ)_max_ = 0.001
173 parameters	Δρ_max_ = 0.82 e Å^−^^3^
0 restraints	Δρ_min_ = −0.37 e Å^−^^3^

### Special details

**Table:**

Geometry. Bond distances, angles *etc*. have been calculated using the rounded fractional coordinates. All su's are estimated from the variances of the (full) variance-covariance matrix. The cell e.s.d.'s are taken into account in the estimation of distances, angles and torsion angles
Refinement. Refinement of *F*^2^ against ALL reflections. The weighted *R*-factor *wR* and goodness of fit *S* are based on *F*^2^, conventional *R*-factors *R* are based on *F*, with *F* set to zero for negative *F*^2^. The threshold expression of *F*^2^ > 2σ(*F*^2^) is used only for calculating *R*-factors(gt) *etc*. and is not relevant to the choice of reflections for refinement. *R*-factors based on *F*^2^ are statistically about twice as large as those based on *F*, and *R*- factors based on ALL data will be even larger.

### Fractional atomic coordinates and isotropic or equivalent isotropic displacement parameters (Å^2^)

**Table:**

	*x*	*y*	*z*	*U*_iso_*/*U*_eq_	
S2	0.18125 (14)	0.34721 (13)	0.46715 (10)	0.0405 (3)	
F4	0.7221 (6)	0.3280 (5)	−0.0587 (3)	0.0829 (16)	
O15	0.7912 (4)	−0.1016 (4)	0.3039 (3)	0.0520 (12)	
N1	0.2057 (5)	0.0276 (4)	0.3682 (3)	0.0387 (10)	
N3	0.4983 (5)	0.2512 (4)	0.4147 (3)	0.0349 (10)	
C2	0.3046 (5)	0.2035 (5)	0.4140 (3)	0.0351 (11)	
C4	0.6032 (5)	0.1414 (5)	0.3474 (3)	0.0344 (11)	
C5	0.4912 (5)	−0.0550 (5)	0.3312 (3)	0.0346 (11)	
C6	0.2961 (6)	−0.1043 (5)	0.3369 (3)	0.0352 (11)	
C15	0.6152 (6)	−0.1729 (5)	0.3037 (4)	0.0394 (14)	
C16	0.5357 (6)	−0.3717 (6)	0.2740 (4)	0.0455 (14)	
C41	0.6337 (6)	0.1890 (5)	0.2361 (4)	0.0380 (11)	
C42	0.4755 (7)	0.1672 (6)	0.1611 (4)	0.0450 (14)	
C43	0.5034 (8)	0.2138 (7)	0.0611 (4)	0.0521 (17)	
C44	0.6923 (9)	0.2814 (7)	0.0386 (4)	0.0581 (17)	
C45	0.8513 (8)	0.3036 (7)	0.1095 (5)	0.0604 (17)	
C46	0.8225 (7)	0.2573 (6)	0.2092 (4)	0.0473 (14)	
C61	0.1533 (5)	−0.2885 (5)	0.3148 (4)	0.0403 (13)	
H1	0.087 (7)	0.005 (6)	0.370 (4)	0.036 (12)*	
H3	0.572 (8)	0.356 (8)	0.446 (4)	0.051 (14)*	
H4	0.73478	0.16638	0.38822	0.0414*	
H16A	0.45343	−0.41343	0.20285	0.0679*	
H16B	0.45700	−0.40981	0.33158	0.0679*	
H16C	0.64443	−0.42238	0.26819	0.0679*	
H42	0.34597	0.11967	0.17837	0.0543*	
H43	0.39509	0.19929	0.00998	0.0629*	
H45	0.98007	0.35005	0.09089	0.0727*	
H46	0.93223	0.27237	0.25940	0.0573*	
H61A	0.15509	−0.35405	0.23847	0.0604*	
H61B	0.02124	−0.28040	0.32542	0.0604*	
H61C	0.19019	−0.35119	0.36620	0.0604*	

### Atomic displacement parameters (Å^2^)

**Table:**

	*U*^11^	*U*^22^	*U*^33^	*U*^12^	*U*^13^	*U*^23^
S2	0.0293 (5)	0.0314 (5)	0.0635 (7)	0.0129 (4)	0.0089 (4)	0.0121 (4)
F4	0.093 (3)	0.086 (3)	0.062 (2)	0.003 (2)	0.0100 (18)	0.0354 (18)
O15	0.0259 (14)	0.0370 (16)	0.099 (3)	0.0146 (12)	0.0106 (15)	0.0218 (16)
N1	0.0226 (16)	0.0336 (17)	0.061 (2)	0.0104 (13)	0.0064 (14)	0.0118 (15)
N3	0.0276 (16)	0.0267 (16)	0.052 (2)	0.0093 (13)	0.0063 (14)	0.0114 (14)
C2	0.0295 (18)	0.0285 (18)	0.051 (2)	0.0105 (15)	0.0064 (16)	0.0149 (16)
C4	0.0245 (17)	0.0282 (18)	0.053 (2)	0.0101 (14)	0.0051 (15)	0.0127 (16)
C5	0.0275 (18)	0.0242 (17)	0.054 (2)	0.0083 (14)	0.0037 (15)	0.0133 (15)
C6	0.0305 (18)	0.0291 (18)	0.050 (2)	0.0115 (15)	0.0048 (15)	0.0148 (16)
C15	0.0275 (19)	0.040 (2)	0.059 (3)	0.0162 (16)	0.0079 (17)	0.0208 (18)
C16	0.033 (2)	0.038 (2)	0.072 (3)	0.0183 (17)	0.0082 (19)	0.017 (2)
C41	0.037 (2)	0.0273 (18)	0.054 (2)	0.0138 (15)	0.0089 (17)	0.0129 (16)
C42	0.039 (2)	0.043 (2)	0.056 (3)	0.0142 (18)	0.0075 (19)	0.0161 (19)
C43	0.056 (3)	0.049 (3)	0.053 (3)	0.017 (2)	0.000 (2)	0.016 (2)
C44	0.073 (3)	0.051 (3)	0.047 (3)	0.010 (2)	0.011 (2)	0.017 (2)
C45	0.055 (3)	0.059 (3)	0.059 (3)	0.001 (2)	0.015 (2)	0.019 (2)
C46	0.037 (2)	0.046 (2)	0.057 (3)	0.0072 (18)	0.0087 (19)	0.016 (2)
C61	0.0263 (18)	0.0289 (19)	0.067 (3)	0.0078 (15)	0.0098 (17)	0.0148 (18)

### Geometric parameters (Å, º)

**Table:**

S2—C2	1.692 (4)	C41—C42	1.390 (7)
F4—C44	1.360 (6)	C42—C43	1.392 (7)
O15—C15	1.226 (5)	C43—C44	1.372 (9)
N1—C2	1.366 (5)	C44—C45	1.363 (8)
N1—C6	1.395 (5)	C45—C46	1.388 (8)
N3—C2	1.329 (5)	C4—H4	1.0000
N3—C4	1.466 (5)	C16—H16A	0.9800
N1—H1	0.82 (5)	C16—H16B	0.9800
N3—H3	0.85 (6)	C16—H16C	0.9800
C4—C41	1.534 (6)	C42—H42	0.9500
C4—C5	1.522 (6)	C43—H43	0.9500
C5—C6	1.349 (6)	C45—H45	0.9500
C5—C15	1.479 (6)	C46—H46	0.9500
C6—C61	1.501 (6)	C61—H61A	0.9800
C15—C16	1.494 (6)	C61—H61B	0.9800
C41—C46	1.391 (7)	C61—H61C	0.9800
			
C2—N1—C6	124.1 (4)	C43—C44—C45	122.8 (5)
C2—N3—C4	124.0 (3)	F4—C44—C45	118.6 (6)
C2—N1—H1	113 (3)	C44—C45—C46	119.1 (5)
C6—N1—H1	123 (3)	C41—C46—C45	120.3 (5)
C2—N3—H3	123 (4)	N3—C4—H4	108.00
C4—N3—H3	113 (4)	C5—C4—H4	108.00
S2—C2—N3	123.5 (3)	C41—C4—H4	108.00
N1—C2—N3	116.3 (3)	C15—C16—H16A	110.00
S2—C2—N1	120.3 (3)	C15—C16—H16B	109.00
N3—C4—C5	109.7 (3)	C15—C16—H16C	109.00
N3—C4—C41	110.6 (3)	H16A—C16—H16B	109.00
C5—C4—C41	111.5 (3)	H16A—C16—H16C	109.00
C4—C5—C15	113.3 (3)	H16B—C16—H16C	109.00
C6—C5—C15	127.3 (4)	C41—C42—H42	119.00
C4—C5—C6	119.3 (3)	C43—C42—H42	119.00
N1—C6—C5	118.9 (4)	C42—C43—H43	121.00
N1—C6—C61	112.2 (4)	C44—C43—H43	121.00
C5—C6—C61	128.9 (4)	C44—C45—H45	120.00
C5—C15—C16	123.3 (4)	C46—C45—H45	120.00
O15—C15—C5	117.4 (4)	C41—C46—H46	120.00
O15—C15—C16	119.3 (4)	C45—C46—H46	120.00
C4—C41—C46	120.0 (4)	C6—C61—H61A	109.00
C42—C41—C46	118.8 (4)	C6—C61—H61B	109.00
C4—C41—C42	121.2 (4)	C6—C61—H61C	109.00
C41—C42—C43	121.2 (5)	H61A—C61—H61B	109.00
C42—C43—C44	117.9 (5)	H61A—C61—H61C	110.00
F4—C44—C43	118.6 (5)	H61B—C61—H61C	109.00
			
C6—N1—C2—S2	−170.5 (3)	C4—C5—C6—C61	174.5 (4)
C6—N1—C2—N3	8.8 (6)	C15—C5—C6—N1	177.7 (4)
C2—N1—C6—C5	−13.1 (6)	C15—C5—C6—C61	−1.6 (7)
C2—N1—C6—C61	166.4 (4)	C4—C5—C15—O15	4.4 (6)
C4—N3—C2—S2	−165.1 (3)	C4—C5—C15—C16	−174.3 (4)
C4—N3—C2—N1	15.6 (5)	C6—C5—C15—O15	−179.3 (4)
C2—N3—C4—C5	−31.5 (5)	C6—C5—C15—C16	1.9 (7)
C2—N3—C4—C41	91.8 (4)	C4—C41—C42—C43	178.7 (4)
N3—C4—C5—C6	25.6 (5)	C46—C41—C42—C43	−0.6 (7)
N3—C4—C5—C15	−157.8 (3)	C4—C41—C46—C45	−178.9 (4)
C41—C4—C5—C6	−97.2 (4)	C42—C41—C46—C45	0.4 (7)
C41—C4—C5—C15	79.4 (4)	C41—C42—C43—C44	0.3 (8)
N3—C4—C41—C42	−61.6 (5)	C42—C43—C44—F4	−179.9 (5)
N3—C4—C41—C46	117.7 (4)	C42—C43—C44—C45	0.2 (8)
C5—C4—C41—C42	60.6 (5)	F4—C44—C45—C46	179.7 (5)
C5—C4—C41—C46	−120.1 (4)	C43—C44—C45—C46	−0.4 (9)
C4—C5—C6—N1	−6.2 (5)	C44—C45—C46—C41	0.1 (8)

### Hydrogen-bond geometry (Å, º)

Cg2 is the centroid of the C41–C46 benzene ring.

**Table:**

*D*—H···*A*	*D*—H	H···*A*	*D*···*A*	*D*—H···*A*
N1—H1···O15^i^	0.82 (5)	2.10 (5)	2.867 (5)	156 (5)
N3—H3···S2^ii^	0.85 (6)	2.50 (6)	3.350 (4)	174 (6)
C16—H16*A*···F4^iii^	0.98	2.50	3.358 (6)	146
C61—H61*A*···F4^iii^	0.98	2.47	3.319 (6)	145
C61—H61*B*···O15^i^	0.98	2.54	3.383 (5)	144
C16—H16*C*···*Cg*2^iv^	0.98	2.92	3.633 (5)	130

Symmetry codes: (i) *x*−1, *y*, *z*; (ii) −*x*+1, −*y*+1, −*z*+1; (iii) −*x*+1, −*y*, −*z*; (iv) *x*, *y*−1, *z*.
